# CNV Workshop: an integrated platform for high-throughput copy number variation discovery and clinical diagnostics

**DOI:** 10.1186/1471-2105-11-74

**Published:** 2010-02-04

**Authors:** Xiaowu Gai, Juan C Perin, Kevin Murphy, Ryan O'Hara, Monica D'arcy, Adam Wenocur, Hongbo M Xie, Eric F Rappaport, Tamim H Shaikh, Peter S White

**Affiliations:** 1 Center for Biomedical Informatics, The Children's Hospital of Philadelphia, Philadelphia, PA, 19104, USA; 2 Division of Oncology, The Children's Hospital of Philadelphia, Philadelphia, PA, 19104, USA; 3 Children's Hospital of Philadelphia Research Institute, Philadelphia, PA, 19104, USA; 4 Department of Pediatrics, University of Pennsylvania School of Medicine, Philadelphia, PA, 19104, USA; 5 Division of Genetics, The Children's Hospital of Philadelphia, Philadelphia, PA, 19104, USA

## Abstract

**Background:**

Recent studies have shown that copy number variations (CNVs) are frequent in higher eukaryotes and associated with a substantial portion of inherited and acquired risk for various human diseases. The increasing availability of high-resolution genome surveillance platforms provides opportunity for rapidly assessing research and clinical samples for CNV content, as well as for determining the potential pathogenicity of identified variants. However, few informatics tools for accurate and efficient CNV detection and assessment currently exist.

**Results:**

We developed a suite of software tools and resources (CNV Workshop) for automated, genome-wide CNV detection from a variety of SNP array platforms. CNV Workshop includes three major components: detection, annotation, and presentation of structural variants from genome array data. CNV detection utilizes a robust and genotype-specific extension of the Circular Binary Segmentation algorithm, and the use of additional detection algorithms is supported. Predicted CNVs are captured in a MySQL database that supports cohort-based projects and incorporates a secure user authentication layer and user/admin roles. To assist with determination of pathogenicity, detected CNVs are also annotated automatically for gene content, known disease loci, and gene-based literature references. Results are easily queried, sorted, filtered, and visualized via a web-based presentation layer that includes a GBrowse-based graphical representation of CNV content and relevant public data, integration with the UCSC Genome Browser, and tabular displays of genomic attributes for each CNV.

**Conclusions:**

To our knowledge, CNV Workshop represents the first cohesive and convenient platform for detection, annotation, and assessment of the biological and clinical significance of structural variants. CNV Workshop has been successfully utilized for assessment of genomic variation in healthy individuals and disease cohorts and is an ideal platform for coordinating multiple associated projects.

**Availability and Implementation:**

Available on the web at: http://sourceforge.net/projects/cnv

## Background

Genome copy number changes (copy number variations, or CNVs) include inherited, *de novo*, and somatically acquired deviations from a diploid state within a particular chromosome segment. CNVs likely contribute substantially to inherited and/or acquired risk for a variety of human diseases, including cancer and neuropsychiatric disorders [1,2]. In addition, CNVs are widely distributed in the genomes of apparently healthy individuals and thus constitute significant amounts of population-based genomic variation [3-8]. New genotyping technologies such as SNP-based arrays provide high-resolution coverage of entire genomes as well as an opportunity for rapidly determining CNV content in sample collections of interest [4,6,7,9-11]. Accordingly, numerous recent studies have described constellations of structural variants in various healthy and disease cohorts [1,2,12,13]. However, interpretation of the exact extent, character, distribution, and effect of these CNVs has been limited by the emerging nature of computational methods for accurate detection, and further challenged by the difficulty in assessing the biological importance of particular CNVs in context with other genomic features and study findings.

Detection of CNVs in high-density SNP arrays requires genotypes that yield high quality intensity and, optimally, allelic ratio data for each locus surveyed. A number of algorithms have been utilized for the detection of CNVs from such genotyping data sets. Software from array vendors such as Illumina and Affymetrix provide basic CNV calls along with graphical interfaces that allow visual inspection of a region of interest. However, these tools generally lack the ability to quickly manage, annotate, and assess CNVs from a sizable number of samples. Moreover, visual inspection becomes challenging for interpreting small or complex rearrangements, or CNVs predicted from genome array data of marginal quality. A number of 3^rd ^party commercial and open-source algorithms, including QuantiSNP [14] and PennCNV [15], utilize algorithms employing Hidden Markov Models [16] to predict CNVs, and these approaches have been developed and adopted for a number of recent genome-wide studies of structural variation. Equally promising are segmentation algorithms such as GLAD [17] and Circular Binary Segmentation (CBS) [18] that have been successfully applied for analysis of data from array-based comparative genomic hybridization (aCGH) platforms. These segmentation approaches are particularly attractive as they have been shown to outperform certain HMM-based approaches for aCGH data [19,20]. Regardless of the approach, these algorithms typically overcall CNV events [12,15,21,22], thus requiring post-prediction methods that consider data quality metrics for distinguishing true events from false positives. Currently, researchers interested in analyzing genotypes for CNV content for the first time, or in setting up production systems for high-throughput analysis and interpretation, are challenged by the considerable variety and limited scope of most existing methods and tools. This is especially true in the use of SNP arrays for clinical diagnostic applications, where reliability and performance are of critical importance.

At the same time, assessing the importance of particular CNVs in context with other genomic features and study findings is a complex task even without robust quality assessment of CNV predictions, especially given limited current knowledge of the distributions of CNVs across the genome and in populations. Contextual genomic and phenotypic annotations need to be considered, while projects involving sizable cohorts also require an infrastructure for managing, accessing, batch-processing, and visualizing annotated CNV predictions.

To address these challenges, we describe the integrated platform CNV Workshop. This package incorporates a modified segmentation algorithm that we have previously applied successfully for detecting pathogenic CNVs in large-scale research and clinical projects [12,13]. CNV Workshop includes a database layer, role-based security and authentication schemes suitable for clinical diagnostic environments, a web-based presentation layer providing textual and graphical visualization of CNV predictions, and integration of CNV content with known genomic and biomedical annotations for rapidly determining the significance of a particular CNV. These components are modular yet seamlessly integrated and together provide an effective platform for identification of high-throughput copy number variation; discovery of inherited, *de novo*, and somatically acquired pathogenic variants; and clinical diagnostics.

## Implementation

### Approach

Conceptually, CNV detection from genotyping data sets consists of two major steps: 1) segmenting chromosome-arrayed genotypes into discrete regions, with probes in each region presenting different signal intensity patterns than adjacent regions; and 2) labeling particular segments that are inherently different in copy number from expected. To accurately predict CNV events, an algorithm requires sufficient sensitivity to distinguish true chromosome copy number state changes from local signal fluctuations.

For aCGH data, these algorithms rely solely upon normalized probe signal intensities, typically log2 ratios of intensity, for segment delineation. Examples include the GLAD and CBS algorithms [23,24]. Genotyping arrays provide an additional useful metric, the allelic ratio, which can be utilized for assessing the copy number state of each segment. Allelic ratio is a measure of the relative signal intensities for probes measuring each of the two alleles at a SNP locus. Besides overall signal intensity at a SNP locus, allelic ratios of a region of true copy number change should present a pattern consistently different from a diploid region. For these reasons, we devised a generic three-step CNV detection methodology that can be applied to all genotyping platforms, with only slight variations required to address platform-specific properties: segmentation, calculation of genotype-specific statistics, and CNV determination. We describe here our implementation and modifications to CBS first for the Illumina Infinium array platform and then modifications required for use with Affymetrix, other SNP, and aCGH arrays.

### Segmentation

In the Illumina Infinium assay, two different probe sets are used to measure the presence of the two different alleles for a given SNP. Allele-specific signal intensities are first normalized into R_subject _values. R_expected _values are then calculated through linear interpolation of the R values for each canonical cluster; the log2 ratio of R_subject _and R_expected _is named the Log R Ratio (LRR) [25]. LRRs above or below zero indicate possible duplication or deletion at a locus, with the degree of deviation correlating with the likelihood of a copy number change. To identify segments of adjacent loci (SNPs) that display an overall LRR pattern consistently distinct from neighboring segments, CNV Workshop implements the segmentation algorithm CBS as its default detection method. However, other segmentation algorithms can be used in place of CBS with only minor source code modifications.

### Additional statistics

After the segmentation step, additional LRR and allelic ratio (B-allele frequency, or BAF) statistics are then calculated for each segment, which are critical for ensuring high quality CNV determinations. The results are then stored in a MySQL database along with the chromosomal coordinates of each segment. For LRRs, two simple statistics are calculated: standard deviation of LRRs by sample and by chromosome, and mean LRR for each chromosome and each segment. Similarly for BAF, three statistics are devised and calculated for each segment: percentage of SNPs with BAFs between 0.6 and 0.4, b2.sd (Equation 1), and b3.sd (Equation 2). These statistics are designed as straightforward measures of the distribution pattern of the BAFs in a segment. For b2.sd and b3.sd, X_i _represents the BAF for the ith SNP of the segment and n represents the total number of SNPs in the segment.(1)

For b2.sd, the constants chosen for the equations are expected BAF values for SNPs in a normal diploid segment for the homozygous AA alleles (0), homozygous BB alleles (1), and heterozygous AB alleles (0.5). For the b3.sd, the constants are the expected values of SNP BAFs in a monoallelic duplication: AAA (0), BBB (1), ABB (0.67), and AAB (0.33) [25]. In this way, b2.sd is expected to be significantly smaller than b3.sd when the segment is truly diploid, and the opposite is expected when the segment is a duplicated or amplified region.

### CNV determination

The likelihood of a segment being predicted as a CNV is determined by many attributes of the segment, especially the BAF statistics, the mean LRR, and the number of SNPs. Although copy number determination can be performed directly after the segmentation step, delay of the copy number calling step affords greater flexibility. Subsequent use of a modified set of criteria for calling copy number changes will not require the repeat of the segmentation step, which is much improved but still computationally intensive with the current implementation of the CBS algorithm. However, the values to use might need to be changed based on the goal of the analysis and the nature of the samples of interest. We have previously reported CNV Workshop threshold values for calling germline heterozygous deletions, homozygous deletions, and duplications from Illumina 550 K data that we found effective for a wide range of samples and genotype quality scores [12,13]. An effective way for learning a new platform and developing appropriate threshold values is by taking advantage of the widely available and validated CNV contents of the 270 HapMap samples, as well as the genotyping data of these same samples from different platform vendors. HapMap data for a new platform can first be processed with CNV Workshop. Thresholds that provide desirable or acceptable Type I and Type II rates can then be obtained by comparing calls derived using different thresholds for known variants. Using this process, we have adopted the algorithm for a number of genotyping platforms, including Illumina 610-Quad, 660-Quad, and Affymetrix 6.0 arrays (thresholds available at: http://cnv.sourceforge.net/).

A number of variables in addition to the array platform, including the particular samples and the reference group used for allele calling, may influence the set of parameters that will function optimally in a given setting. For example, tumor samples are often hyper- or hypodiploid across a genome or certain chromosomes. Commonly employed global normalization algorithms often assume that the majority of probe intensities should remain at a diploid state and do not incorporate *a priori *methods for inferring degree of aneuploidy. CNV Workshop provides a convenient mechanism for determining the existence and degree of hyper- or hypodiploidy. As b2.sd and b3.sd statistics are calculated for each chromosome, highly skewed chromosome-specific b2.sd/b3.sd ratios indicate chromosome-level aneuploidy. However, normalized values of a hyper- or hypodiploid sample are also skewed due to global normalization; thus, detection of copy number changes at higher resolution requires cutoff mean intensity values of a segment to be adjusted accordingly for tumor samples. We advise users to experiment with these parameters as appropriate. To assist with this process, an "advanced search" function has been included in CNV Workshop for adjusting these parameters. In addition to these criteria, segments can be queried based on physical size and number of SNPs.

### Affymetrix arrays

Affymetrix genotyping arrays are widely used for copy number variation detection. Similar to Illumina's LRR metric, the Affymetrix Genotyping Console application, as well as commercial packages such as Partek Genomics Suite, calculate log-transformed ratios (Log2 ratios) of summarized probe intensities for a SNP of a given sample, as compared to the same intensities measured in control samples [26]. Additionally, these packages provide allelic ratios comparable to BAFs. We have successfully used CNV Workshop to analyze Affymetrix array data by substituting Log R Ratios and B Allele Frequencies with normalized log2 ratios and allelic ratios derived from Partek Genomics Suite. As log2 ratios exhibit greater variance than LRRs and vary across different Affymetrix platforms, different threshold values are required. Certain Affymetrix platforms such as the 6.0 platform incorporate non-SNP copy number probes in addition to SNP probes. While the data from these intensity-only probes is less reliable for CNV determination, the added advantage of increased resolution may be desirable for certain applications. Inclusion of intensity-only probe data is enabled by uploading an additional text file containing intensity values for these probes.

### aCGH and other SNP platforms

CNV Workshop can also be adapted for use with aCGH and other SNP platforms. For aCGH platforms, normalized probe signal intensities, which are typically transformed as log2 ratios of probe intensities of a sample versus controls, are the only available metric for assessing relative copy number. After the segmentation step, the likelihood of a given segment representing a true copy number loss or gain is proportional to the segment mean signal intensity relative to other segments from the same chromosome, or across the entire genome. For this reason, CNV Workshop calculates and stores all probe and segment means, medians, and standard deviations. This information, even in the absence of allelic ratio data, can be used to establish a dynamic yet robust threshold for aCGH data. For example, a segment with mean signal intensity deviating by three standard deviations from the mean signal intensity of all segments is likely to indicate true gain or loss.

### Algorithm performance

**Figure 1:**
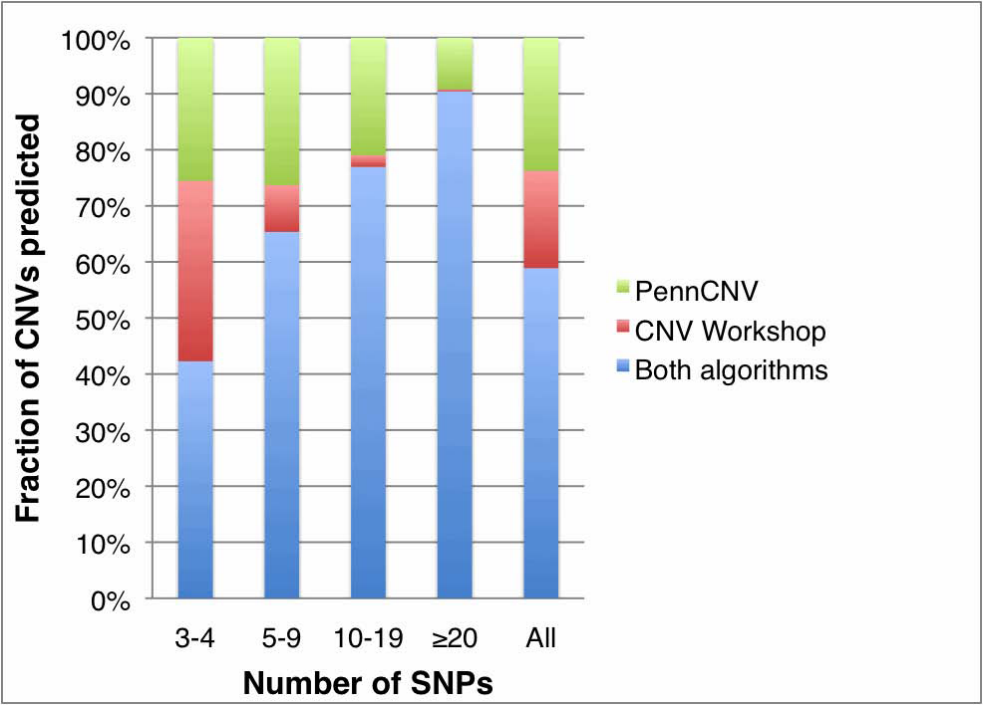
**Comparison of CNV Workshop and PennCNV variant prediction sets**. Depicted is a composite graph showing the fraction of CNVs predicted solely by PennCNV, CNV Workshop, and both algorithms. Each column indicates the fraction of predicted CNVs for a certain size range, and for all CNVs combined (leftmost column).

Direct comparison of CNV detection algorithms is challenging in the absence of a sizable evaluation standard. However, to provide a general measure of algorithm performance, we compared results from CNV Workshop with PennCNV, a commonly used, HMM-based CNV detection algorithm. A set of 112 unique HapMap samples genotyped on the Illumina 550 kv1 platform was analyzed for CNV content using default settings and threshold values for each algorithm (Figure 1). Overall, CNV Workshop and PennCNV were generally concordant (77.5% and 69.0%, respectively). Concordance rate increased substantially as a function of CNV size, but considerable discordance was observed especially for CNVs spanning <5 SNPs. These results indicate some combined contribution of Type II error from CNV Workshop and Type I error from PennCNV for smaller predicted CNVs. Notably, the number of CNVs predicted by PennCNV per sample was inversely proportional to sample-wide LRR standard deviation, but this trend was not observed for CNV Workshop within LRR standard deviation ranges we consider acceptable for analysis (<0.35).

### Architecture

CNV Workshop builds upon a number of open source applications and libraries. The major components of CNV Workshop are a set of scripts for processing the genotyping data, a set of scripts for predicting copy variations and subsequently annotating each CNV, a MySQL database server, a web server hosting a custom instance of the GMOD Generic Genome Browser [27] via CGI, and an Apache Tomcat server hosting the Java-based CNV Workshop web application. These components may reside on the same or different physical computers running either Windows or UNIX-based operating systems such as Linux and Macintosh OS X. As such, the application is well suited to support a set of investigators and projects distributed across an organization or multi-site collaboration. CNV Workshop is best administered by bioinformaticists or computer system administrators on behalf of biologists. However, we also make available a pre-installed virtual machine (Linux CentOS 5.4) to ease installation for those with a powerful computer and virtualization software such as Parallels, VMWare, VirtualBox, or Xen.

### Data processing and management

Raw genotyping data are first processed with an R script that automatically segments based on the SNP intensity data, calculates additional statistics, and subsequently inserts the segment information and these statistics into a MySQL database. In our setting, segmentation of data from a single Illumina 550 k array, using an Intel Xeon 3.16 GHz server running Centos 5 with 16 GB of RAM, required 18 minutes and less than 1 GM RAM on a single CPU core. A Perl script then analyzes the segment data files, predicts CNVs, and populates the database with CNV calls. Alternatively, CNV predictions using custom parameter values can be made on-the-fly for specified datasets via the advanced search function in the web application. CNV data sets established by a user are then made visible via the CNV Workshop web application. The database supports the ability to view and manipulate CNVs at the event, sample, and sample cohort levels.

### CNV annotations

CNVs are automatically annotated for genotyping and genome-derived metadata, including CNV type (e.g., deletion or duplication), number of SNPs in an event, genomic sequence position, and quality metrics. A database parameter specifies genome build version such that annotations reflected in CNV Workshop are accurate with respect to build. The default value is build hg18 as most array platforms are currently based on this build. Additional automated annotations include gene content, association with known disease loci or genes, and overlapping public CNVs from the Database of Genomic Variants (DGV) [4]. This is accomplished by running programs that query remote data sources such as DGV and UCSC Known Genes, certain of which are cached locally for performance reasons. CNV Workshop also comes pre-loaded with the CHOP CNV collection from 2,026 healthy controls [12]. An optional custom track is reserved such that a set of customized annotations can be readily incorporated. To facilitate this function, the site administrator is able to load into the database a mapping of annotation labels to loci. These labels are displayed in both the graphical and tabular presentations for CNVs that overlap the annotated loci. For instance, the custom track might be used to flag CNVs that overlap genes in a specific pathway or are associated with a disease of particular interest to the research group.

### Administration

Analysis and loading of data sets into the database, along with creation and updating of local mirrors of annotation sources, is accomplished by the execution of programs on the command line. Through the Admin tab of the web application, an administrator can assign role-based privileges so that access to a data set is restricted to a group of users. This function is controlled by the creation, deletion, and modification of three entities: users, groups, and data sets. Users have three attributes: email address, password, and group membership. Groups are essentially many-to-many mappings between users and data sets. Finally, administrators use the Admin interface to provision data sets that have previously been loaded into the database.

## Results

CNV Workshop's web application allows users to flexibly query CNV data sets, view annotated search results, mark and save subsets of queries in their accounts, and download query results in a variety of formats.

### Queries

**Figure 2:**
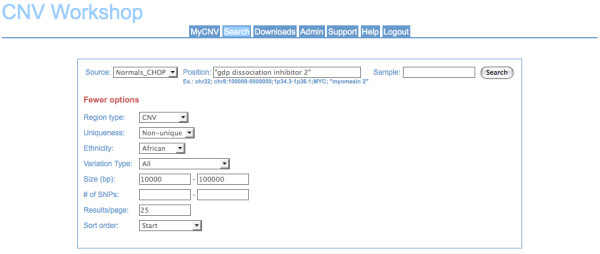
**CNV Workshop query interface**. Screenshot of the simple query interface in "annotated" mode for the CHOP CNV map database. Annotated mode enables a user to query by sample. Positional queries supported are chromosome(s), cytogenetic band(s), sequence position or range(s), and gene name(s). Most non-standard gene names are recognized and normalized to HGNC gene symbols. For the CHOP CNV map, additional searchable fields are CNV type (CNV, CNV region, or CNV block), ethnicity, and uniqueness (unique or non-unique/observed in multiple unrelated individuals).

**Figure 3:**
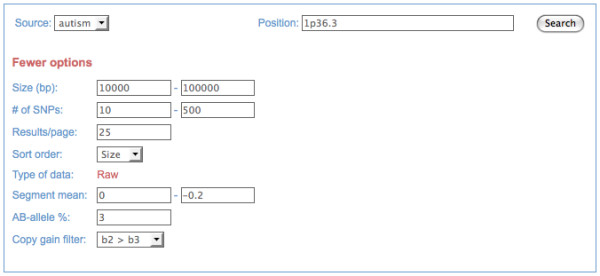
**Screenshot of the advanced query interface in "raw" mode for a database of autism samples**. Raw mode enables a user to query by segment mean, minor (AB) allele frequency, and to filter results based upon allelic ratio statistics.

For each data set, users can choose from a basic search function that queries CNV predictions and annotations of these CNVs (Figure 2), or an advanced search function that queries segmented data prior to CNV determination (Figure 3). For both data types, supported search parameters include genomic position (chromosome, cytogenetic band or band range, sequence position range, or gene name), variation type (duplication, heterozygous deletion, or homozygous deletion), and CNV size (base pairs or number of SNPs). For the advanced search function, additional supported queries include segment mean, heterozygous allele frequencies, and copy gain filter, which allows a user to set parameters for establishing customized CNV detection thresholds.

### Presentation

**Figure 4:**
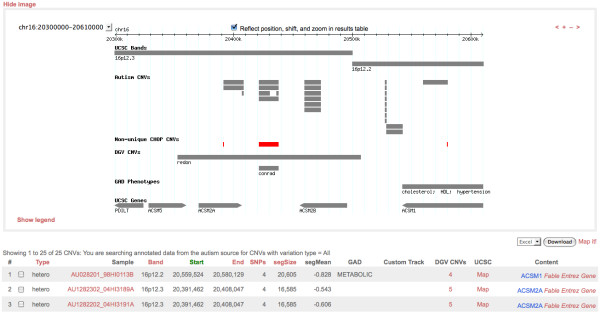
**Presentation of query results in CNV Workshop**. Depicted are results for a chromosome 16 query of sequence position range 20,300,000-20,610,000 in an autism cohort. Top panel: graphical display. Layers (top to bottom) represent the sequence position (top), cytogenetic bands, CNVs observed in the autism cohort, CNVs in the CHOP CNV map, CNVs in the Database of Genomic Variants (labels indicate the study), phenotypes of Genetic Association Database studies, and UCSC Known Genes. All glyphs hyperlink to corresponding database records. Bottom panel: tabular display for a subset of CNVs. Sortable column headers are colored red or (for current sort order) green. Each row value colored red denotes a hyperlink to a corresponding external database record. Checkboxes at far left allow a user to save certain CNVs in the MyCNV clipboard.

Query results are presented to a user both graphically and in a tabular format (Figure 4). The graphical image is rendered via a GMOD Generic Genome Browser (GBrowse) display. The GBrowse layer presents project-specific CNVs in one or more regions of interest as a custom track, along with default tracks for the healthy control set, DGV content, annotations from the Genetic Association Database (GAD) [28], and the Known Genes and cytogenetic band tracks from the UCSC Genome Browser [29]. Queries that yield results for multiple, non-overlapping genomic regions are rendered as separate visualizations, which are viewable by selecting region-specific views from a drop-down list.

The tabular display generally reiterates the graphical display but includes additional features of each CNV in a row-by-row format, including variation type, sample ID, cytogenetic band, sequence position, number of SNPs, segment size in base pairs, and segment mean log R ratio. To facilitate further exploration of particular CNVs, both the graphical and tabular displays include links to the external genomic resources DGV, GAD, and the UCSC Genome Browser. In addition, for CNVs that overlap genes, annotations and hyperlinks are provided to corresponding gene content from NCBI Entrez, Entrez Gene, and MEDLINE-mined literature through FABLE [30,31].

### Saving and downloading

Query results can be downloaded in a variety of formats, including Excel, CSV, XML, and BED. The BED format is especially useful as it is compatible with visualization in the UCSC Genome Browser as well as additional analysis tools such as Galaxy [32]. CNV Workshop also supports the concept of persistent, editable "clipboards" of previous search results through the MyCNV function. Users can create multiple clipboards, each of which can store selections from multiple queries. Clipboards persist across logins until deleted by the user.

## Discussion

Structural variants are increasingly recognized as crucial contributors to genome diversity and disease risk. While many studies exploring associations between structural variants and individual disorders have recently emerged, most human diseases with a genetic component have yet to be systematically investigated. Analysis of new and existing genotype data generated for association studies or clinical purposes will require more robust tools to facilitate these numerous and often large-scale studies. Accordingly, our design of CNV Workshop attempted to address both current impediments to rapid analysis and the need to accommodate a variety of approaches. An additional objective was to incorporate features to facilitate workflow, data management, and data interpretation tasks that are often underappreciated in CNV studies. Finally, we aimed to create a platform that was compatible with both discovery and diagnostic needs.

Current methods for analyzing structural variants are diverse, including only moderately compatible approaches for genotyping, CNV calling, and analysis requirements. This diversity has created challenges for groups or consortia interested in combining data sets derived from multiple platforms or analytical approaches. While CNV Workshop cannot overcome these challenges, there are several features that can assist. First, CNV Workshop supports both Illumina and Affymetrix SNP arrays that constitute the majority of current SNP array data, and it can be readily adapted to other SNP and aCGH platforms with relatively minor effort. Moreover, data sets with pre-existing CNV calls can be uploaded into CNV Workshop for integrated annotation, visualization, and cross-comparison. This feature also provides flexibility for users who wish to use other detection algorithms, although the CNV Workshop architecture enables additional algorithms to be incorporated directly into the workflow with modest effort. Moreover, as CNV calls are locally stored, particular CNVs or samples can be quickly and conveniently re-queried or re-analyzed with differing attribute or parameter settings, especially as new data sets are added incrementally. Finally, for use in diagnostic settings, we have incorporated features such as role-based access control and the ability to view and store CNV content relative to healthy controls.

We have predominantly used the CBS algorithm for the segmentation step, although we have tested all segmentation algorithms described by Lai and colleagues [19] on the Log R Ratios of Illumina genotypes. In terms of sensitivity and specificity, CBS was found to be one of the better performing algorithms by the Lai study. CBS was also the only algorithm that could consistently segment chromosomes correctly for all samples with known CNVs that we tested. Our work led us to an appreciation of including post-segmentation analyses that incorporate quality metrics into the CNV determination process.

We have successfully applied our modified CBS process for analyzing over 15,000 research-derived genotypes spanning more than a dozen pediatric disorders, along with nearly 2,000 clinical samples for diagnostic purposes [12,13]. These efforts have included validation trials using a variety of experimental approaches. Future versions of CNV Workshop plan to exploit newer detection methods, possibly including the simultaneous application of multiple algorithms, as well as approaches that consider additional genomic features.

## Conclusions

As disease-oriented genomic analysis continues to evolve, large-scale array- and sequence-based studies will become increasingly possible. This evolution will likely necessitate more sophisticated analytical, workflow, and data infrastructure elements. CNV Workshop provides a first-generation platform for managing many of the complex tasks required to productively process and assess structural variation content from high-resolution genomic array data. Currently, we are formulating strategies for further accommodating these needs within the CNV Workshop framework. Possible extensions include features to more directly allow cross-cohort comparisons and to assist with clinical diagnostic applications via automated disease labeling and report generation. In addition, we are developing features for viewing regions of homozygosity and labeling potential mosaic CNVs. Finally, we are exploring methods for both expert- and machine-ranking of CNVs to assist the considerable challenge of assessing pathogenicity for structural variants in disease settings.

## Availability and requirements

**• Project name: **CNV Workshop

**• Project home page: **http://sourceforge.net/projects/cnv

**• Operating systems: **Linux or Mac OS X operating systems

**• Programming languages: **Java, R, Perl

**• Other requirements: **Maven 2, Java JDK 6, Perl 5.8.6+, Apache or other web server, Apache Tomcat 6.0, MySQL client and server 4.1 or 5.0, Generic Genome Browser 1. X, R 2.8, GNU Make

**• License: **GNU Affero GPL v3 or any later version

## Authors' contributions

XG and JCP planned, wrote and tested most of the first iteration of software, including algorithm development. KM, RO, AW, and MD substantially contributed code, subsequent aspects of design, testing, and deployment. HX, EFR, and THS contributed user requirements, functionality input, and expert testing. PSW oversaw project development. XG, KM, and PSW wrote and revised the manuscript. All authors read and approved the final manuscript.
